# Out of the ‘host’ box: extreme off-host conditions alter the infectivity and virulence of a parasitic bacterium

**DOI:** 10.1098/rstb.2022.0015

**Published:** 2023-03-27

**Authors:** Enav Marcus, Tal Dagan, Weaam Asli, Frida Ben-Ami

**Affiliations:** School of Zoology, George S. Wise Faculty of Life Sciences, Tel Aviv University, Tel Aviv 6997801, Israel

**Keywords:** climate change, *Daphnia magna*, dryness, *Pasteuria ramosa*, temperature

## Abstract

Disease agents play an important role in the ecology and life history of wild and cultivated populations and communities. While most studies focus on the adaptation of parasites to their hosts, the adaptation of free-living parasite stages to their external (off-host) environment may tell us a lot about the factors that shape the distribution of parasites. *Pasteuria ramosa* is an endoparasitic bacterium of the water flea *Daphnia* with a wide geographical distribution. Its transmission stages rest outside of the host and thus experience varying environmental regimes. We examined the life history of *P. ramosa* populations from four environmental conditions (i.e. groups of habitats): the factorial combinations of summer-dry water bodies or not, and winter-freeze water bodies or not. Our goal was to examine how the combination of winter temperature and summer dryness affects the parasite's ability to attach to its host and to infect it. We subjected samples of the four groups of habitats to temperatures of 20, 33, 46 and 60°C in dry and wet conditions, and exposed a susceptible clone of *Daphnia magna* to the treated spores. We found that spores which had undergone desiccation endured higher temperatures better than spores kept wet, both regarding attachment and subsequent infection. Furthermore, spores treated with heightened temperatures were much less infective and virulent. Even under high temperatures (60°C), exposed spores from all populations were able to attach to the host cuticle, albeit they were unable to establish infection. Our work highlights the sensitivity of a host-free resting stage of a bacterial parasite to the external environment. Long heatwaves and harsh summers, which are becoming more frequent owing to recent climate changes, may therefore pose a problem for parasite survival.

This article is part of the theme issue ‘Infectious disease ecology and evolution in a changing world’.

## 1. Introduction

Parasites play a pivotal role in the ecology and life history of wild and cultivated populations and communities [1,2]. While disease agents have been studied extensively, the COVID-19 pandemic and the rising awareness of climate change emphasize the need to better understand the factors affecting disease outbreaks. Various studies have shown that abiotic factors, predation on free-living stages, host size, age, behaviour and diet can all alter the infectivity, survival, richness and abundance of parasites [3–8]. Studies of the impact of environmental factors on parasites mainly focus on the indirect effects of this impact on the host immune system or intrinsic host–parasite interactions [9–12]. Empirical tests of direct effects on the parasites themselves are scarce and were mostly conducted using ectoparasites, where exposure to the environment is more visible [13–17]. As many endoparasites have at least one off-host stage for horizontal transmission, this stage must survive the external environment, which may differ greatly from the host's internal environment. Furthermore, very few studies have examined the role of abiotic factors on the survival, virulence and infectivity of off-host stages of endoparasites [18]. Not only do we want to learn how the environment affects the parasites, but we are also interested in understanding which limitations are imposed by extreme conditions. This is especially interesting in light of climate change and increased probability for extreme weather conditions, such as droughts, heatwaves, flash floods and storms [19].

Many parasites are exposed to the external environment while waiting to encounter a host, and for certain environmental conditions, they may suffer from damage to the components of the cell, such as outer cell components, cytoplasmic membrane, ribosomal RNA, proteins, enzymes and DNA [20]. Survival time outside the host strongly depends on the parasite: rhinoviruses can survive only up to 3 h on surfaces or skin [21]. The first larval stage of certain trematodes (miracidia) can survive up to 16 h [22,23], and some trematode cercariae (motile stage) can survive up to 48 h [24]. In freshwater bodies, parasite survival times can be months or years, for example the non-culturable (VBNC) form of cholera [25] and the bacterial and microsporidial stages infecting waterfleas (genus *Daphnia*) [26–28]. Survival often depends on the external conditions, such as environmental temperature and humidity. Depending on surface material and environmental conditions, the SARS-CoV-2 virus can survive from a few hours up to 28 days [29,30]. For several other human viruses with survival times of days to weeks, survival depends on ambient temperatures, sunlight, humidity and the frequency of desiccation cycles [31]. Longer survival ability in the external environment is clearly an advantage, as the chances for transmission increase. Indeed, many parasites have evolved specialized structures to resist drying, heating or freezing. Under natural conditions, the well-protected eggs of tapeworms (genus *Taenia*) were reported to survive for up to 12 months [32]. Other such examples are the highly stress-resistant endospores of Firmicute bacteria (e.g. anthrax spores, [33]), the capsules of certain viruses (e.g. occlusion bodies of baculoviruses, [34]) and complex eggshells of some nematodes (e.g. round worm eggs, [35]). These highly specialized structures are adaptations to the ‘external’ environmental conditions. To understand how parasites evolve strategies to increase their survival outside the host, it would be helpful to understand how changing environmental conditions impact parasite survival, by studying within-species variation and the stress-resistance of the environmental stages.

The environmental stages (sometimes called ‘off-host’ stage) in a parasite's life cycle have received little attention with regard to changing climate [36,37]. These stages evolved not only to survive the average climatic conditions, but also to cope with extremes, such as drought, freezing and heat stress, as well as cope with the conditions in which the host can only survive through diapause or by seasonal migration to other habitats (e.g. birds). While the host itself offers the parasite a fairly stable environment, the external environment may be much more variable and challenging, and extreme climatic conditions might present a particular challenge for the off-host stages. Consequently, these stages, more than the within-host stage, will determine the parasite's ability to cope with climate change.

We used the host–parasite system *Daphnia magna*–*Pasteuria ramosa*. Both the host and its bacterial parasite are common in pools and lakes worldwide, and are exposed to highly variable climatic conditions [38–40]. Our aim was to investigate the influence of two crucial environmental factors—winter freezing and summer dryness—on the parasite's transmission stage (=spores). During the infection process, *P. ramosa* undergoes a series of steps, such as attachment to the host cuticle, penetration into the host, and within-host growth [27]. We therefore expected that heat damage could be expressed differently in certain steps of this process, such as penetration into the host, encounter with the immune system, and parasite reproduction and propagation. To test our expectations, we first investigated how winter freezing and summer dryness affect the infectivity, virulence, within-host growth of *P. ramosa* and host reproduction, by treating spores to a variety of temperature and dryness combinations prior to host exposure. Then, we examined the impact of the same factors on the attachment of the parasite to the host. Since this step is a prerequisite for successful infection [27], this step may allow better understanding of the results of the first experiment. By building on our knowledge of the infection process in this system, our novel combined approach allowed us to unravel how these factors interact with the infection steps in determining the outcome of infection.

## 2. Methods

### (a) The study system

*Daphnia magna* is a planktonic filter-feeding freshwater crustacean, with a body length of 0.5–5 mm (juveniles to adults). It can reproduce both asexually, via parthenogenesis, producing genetically identical offspring, and sexually, generating long-lasting resting stages that contain dormant embryos [38]. It has a Holarctic distribution and occupies pools with different climatic characteristics and patterns [41].

*Pasteuria ramosa* is one of the most common disease agents of *D. magna*. It is a gram-positive, bacterial endoparasite that infects species of the genus *Daphnia* and other closely related cladocerans. Similar to its host, *P. ramosa* is widespread in the Holarctic [39,42,43]. The parasite activates in the presence of the host, attaches to the host's oesophagus after being ingested and starts penetrating the cuticle, a process that takes 12 to 36 h to complete. The parasite can be detected using light microscopy about 8 days after host exposure, when it shows its ‘cauliflower’ stage [27]. Each cauliflower will develop into many young grapeseed-shaped spores that will develop to become mature spores. The parasite causes host castration, enlarged host body size (gigantism) and premature death [44]. During the late phases of infection, infected hosts can go through castration relief, during which they produce one or more clonal clutches [45,46]. *Pasteuria ramosa* produces endospores that can survive in a dormant state in the sediment for up to 30 years [27]. Susceptibility of *P. ramosa* to its host is subject to genotype-by-genotype (GxG) interactions [47,48].

#### (i) Infection experiment

We used seven *P. ramosa* isolates that originated from habitats subjected to four dryness conditions: two from habitats that are wet (but not frozen) in winter and dry in summer (WD), two from habitats that are frozen in winter and wet in summer (FW), one from a habitat that is frozen in winter and dry in summer (FD), and two from habitats that are wet (but not frozen) in winter and wet in summer (WW; table 1). Unfortunately, owing to an accidental spore loss, the FW habitat was excluded from this experiment.

**Table 1.** *Pasteuria ramosa* isolates that were used in this study. The environmental conditions from which each clone originated are shown as the presence or absence of dryness during the summer, and the presence or absence of freezing during the winter. Each isolate was classified into one of four groups, based on the conditions during winter and summer: WD, non-frozen dry; WW, non-frozen wet; FD, frozen dry; FW, frozen wet. ‘Experiment’ refers to the experiment the parasitic isolate was used in, I for the infection experiment, A for the attachment experiment. Isolates CH-H-1 and DE-R1 were first included in the infection experiment, but owing to spore loss they were subsequently excluded.

isolate	origin	freeze	dry	group	longitude	latitude	experiment
FI-VIW-1	Finland	yes	yes	FD	23.201	59.830	I, A
BE-KN-2	Belgium	no	no	WW	3.334	51.356	I
GB-EA-24	England	no	no	WW	−2.341	55.702	I, A
CH-H-1	Switzerland	yes	no	FW	8.863	47.558	A
DE-R-1	Germany	yes	no	FW	10.424	54.208	A
DE-G1	Germany	yes	no	FW	10.967	54.282	A
Nitzanim	Israel	no	yes	WD	34.626	31.724	I, A
Tirtza	Israel	no	yes	WD	35.52	32.07	I, A

Each parasite isolate was propagated in cultures of *D. magna* clone HU-HO-2 (Bogarzoto, Hungary), which is susceptible to all *Pasteuria* isolates used in this study. Spores were then kept at −20°C in Eppendorf tubes until use. To examine how different environmental conditions affect *P. ramosa* spores, we used cadavers of infected *Daphnia* (filled with spores) from each of the seven isolates in eight treatments (four  temperatures ×  two dryness conditions). The temperatures selected for the experimental treatments range from ambient laboratory conditions (20°C) to the extreme temperatures that are found during the hot season of the hottest habitat in this study (Tirtza and Nitzanim, Israel) [41]. In order to test the limits of the parasite's spores and to understand the influence of the rising temperatures owing to climate change, the highest temperature in our study (60°C) exceeds the maximum temperature recorded in desert ponds of Israel by 5°C [49]. Furthermore, we used two more temperatures in between (33°C and 46°C) to generate a uniform gradient. For each temperature, we treated spores in dry and in wet conditions. For the wet treatments, spore-filled cadavers of hosts were suspended in 1 ml of artificial medium (ADaM [50]). For the dry treatments, Pasteur pipettes were used to drain out the water, upon which the tubes were kept open for 24 h, until all liquid remnant had evaporated. Temperature treatments were applied using heat blocks at 20, 33, 46 and 60°C for 72 h. Thereafter, we used pestles to crush the spore-filled *Daphnia* cadavers, in order to suspend the parasite spores in the medium. We counted mature spores using a Thoma haemocytometer, at 400x magnitude (DM2500, Leica, Germany), and adjusted spore concentrations. This process lasted 2 days, and each spore suspension was counted twice and averaged. All spores were kept at 20°C during the counting session to avoid extreme temperature changes.

Prior to the experiment, we raised the host mother generation (HU-HO-2 clone) in 200 ml jars filled with ADaM, with 10–15 mothers per jar, and fed them ad libitum with the unicellular algae *Scenedesmus gracilis*. The mothers were kept at 20°C, with a 16 L : 8 D cycle. Six days prior to exposure, we separated 0- to 24 h-old neonates from the mothers and kept them under the same conditions as the mother generation. Two days after the separation from the mothers, each offspring was placed individually in a small jar containing 20 ml of ADaM. On day 5, we used 80 000 spores of each treatment to infect 20 *Daphnia* per treatment (20 *Daphnia* × 8 treatments × 5 isolates = 800 parasite-exposed *Daphnia*). This high number of spores was used to achieve infection in all the viable treatments. We also sham-infected 20 individuals with a placebo suspension (controls). All individuals used were females.

Five days post-exposure, we transferred all *Daphnia* to jars filled with 100 ml of ADaM. We examined the *Daphnia* daily, recorded survival and clutches released, and replaced the medium after the release of a clutch (or once a week if no clutch was released). Each individual was fed daily with increasing amounts of the unicellular algae *S. gracilis* as follows: 1–3 days old 1 × 10^6^, 4–8 days old 2 × 10^6^cells, 9–14 days old 3 × 10^6^cells, 15–17 days old 5 × 10^6^cells, 18–21 days old 6 × 10^6^ cells, 22–26 days old 7 × 10^6^ cells, 27–29 days old 8 × 10^6^ cells, 30–36 days old 9 × 10^6^ cells and greater than 36 days old 10 × 10^6^ cells. Dead animals were collected and stored at −20°C for later spore counting. Eighty-four days post-infection, after all infected animals had died, the remaining *Daphnia* were collected and stored at −20°C. All *Daphnia* were crushed with pestles and screened for infection by taking two samples from each individual. Spores from all maturity stages were counted three times.

### (b) Statistical analysis

We used R (v. 4.1.2) for the analysis and the ‘ggplot2’ package for visualization. We fitted generalized linear models (GLM) to our dependent variables of interest: parasite infectivity, spore production and host reproduction. The independent variables were temperature (continuous), dryness (binary) and habitat group (hereafter referred to as group). In each analysis, we pre-checked the random structure of the ‘parasite isolate’ variable nested within the fixed ‘group’ variable and found that models with no random structure had a lower Akaike information criterion (AIC); thus, no random structure was applied, and the isolates were pooled into groups. The initial models built for the statistical analysis contained all independent variables and all possible interactions among them. Insignificant interactions (*p* > 0.1) were then removed from the models. No further model selection was applied. The significance of the variables was tested using the 'anova' function in the ‘car’ package. *Post hoc* comparisons were calculated using the ‘emmeans' package. For each trait, we carefully selected the model type with the correct distribution. To analyse infectivity (binary), we used logistic binomial regression. For spore production, we used the total amount of spores (summed young and mature spores) and modelled using the common Gamma log-link, which is often used for positive nonlinear data. We also used the Gamma log-link distribution for the survival analysis of infected hosts. The number of clutches produced was modelled with a negative binomial distribution owing to over-dispersion.

#### (i) Attachment experiments

**Figure 1.**
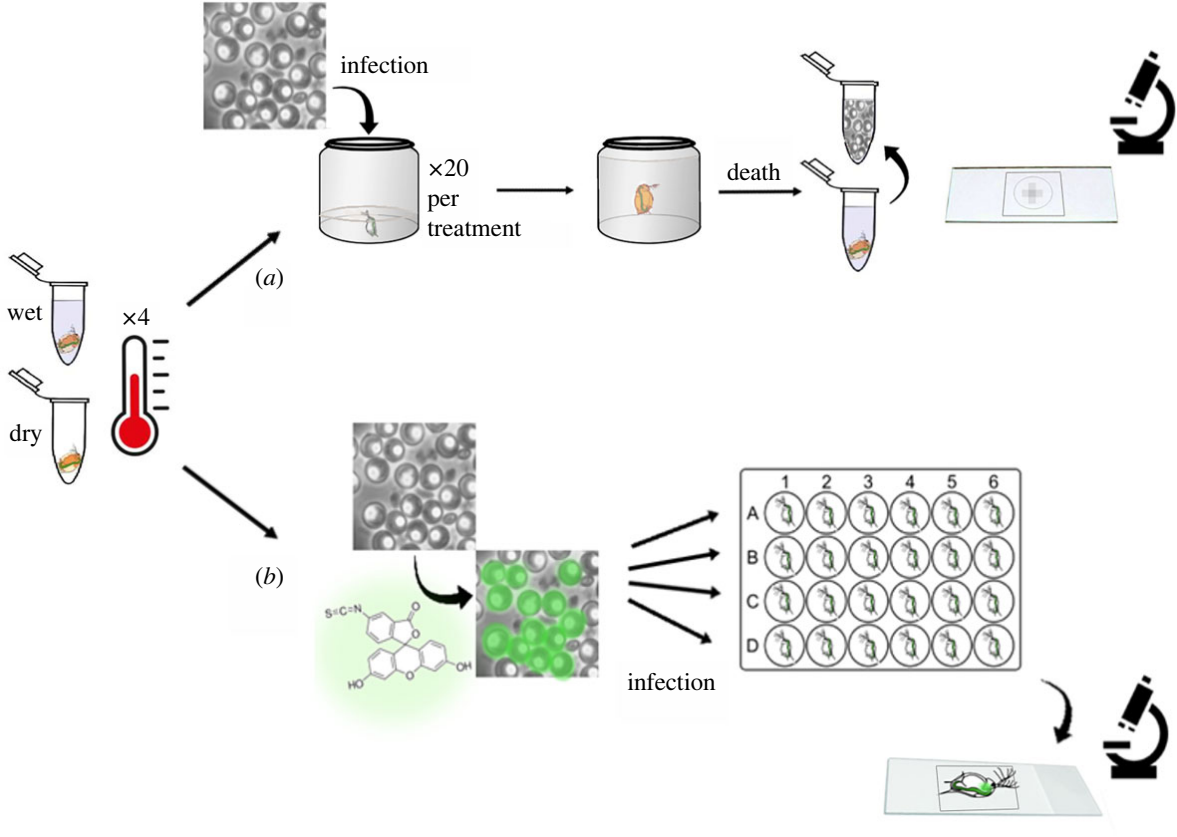
Experimental design of the experiments. Prior to infection, each *P. ramosa* isolate was treated with one of four temperatures: 20, 33, 46 and 60°C, under two dryness conditions: dry and wet. The tubes were incubated for 72 h for the infection experiment (*a*) and kept for 2–3 days at 20°C, after which 20 *Daphnia* were exposed to 80 000 spores each. We raised the *Daphnia* until they died naturally, crushed them and counted the spores. For the attachment experiment (*b*), after incubation, we applied fluorescence labelling to the walls of the spores (attachment test as per Duneau *et al*. [51]). One day after labelling, we exposed *Daphnia* to each spore treatment after singly placing it in 24-well plates with 1 ml of ADaM. For each parasite treatment, we exposed 12 *Daphnia* to 5000 spores for an hour. We counted the number of spores that attached to the host's oesophagus and/or hindgut under fluorescence light and x400 magnification. (Online version in colour.)

**Figure 2.**
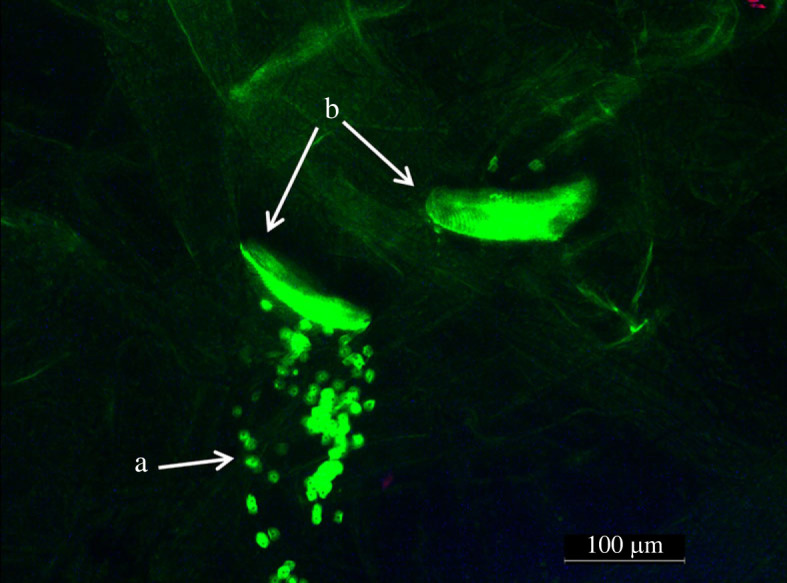
*Daphnia* oesophagus under fluorescence microscopy (after the animal had been squashed): a, labelled spores, and b, the tip of the two auto-fluorescent mandibles used to locate the oesophagus. (Online version in colour.)

In these experiments, we used the same *D. magna* clone and most of the *P. ramosa* isolates used in the infection experiment (table 1). We performed several short-term experiments over a period of 2 years. The course of these experiments and the treatments applied to the spores were identical to the ‘infection experiment’. After applying the appropriate 72 h temperature treatment, we added 0.1 ml of ADaM to the dry treatments and reduced the volume of the wet treatments to 0.1 ml. We then crushed the samples to release the spores and fluorescence-labelled the spores (attachment test as per Duneau *et al*. [51]). One day after labelling, we exposed HU-HO-2 *Daphnia* to all spores. For this, 4- to 8-day-old *Daphnia* were singly placed in 24-well plates with 1 ml of ADaM. The neonates were not fed for 2 days before the experiment, to avoid algae disturbing the examination. For each parasite treatment, we exposed 12 *Daphnia* to 5000 spores. Unlike the amount used in the infection experiment, this number of spores allowed us to count easily and thus notice more delicate changes in the treatments. We left the *Daphnia* with the spores for 1 h in complete darkness at 20°C and then moved each individual to a jar filled with 100 ml of ADaM for 30 min, in order to wash out unattached spores. Then, under fluorescence light and 400x magnification, we counted the number of spores that attached to the host's oesophagus and/or the hindgut (figures 1 and 2). Once we managed to count up to 100 attached spores in an individual, we did not continue to count above 100 spores owing to inaccuracy caused by high spore density. We counted only 10 individuals out of the 12 exposed, as the two spare individuals were used in case a *Daphnia* had moulted before being examined via the microscope (upon moulting attached spores detach from the host with the carapace [52]). Isolates that attached to the hindgut but not to the oesophagus were excluded from the analysis owing to high variability in the number of attached spores within the same treatment, which is known for hindgut attachment [53].

### (c) Statistical analysis

Once 100 spores attached to the *Daphnia*'s oesophagus, it was considered saturated as we know that under standard exposure, the *Daphnia* would have become infected. Below 100 spores, we used the number of spores as an estimate of the attachment rate. For example, if 30 spores were counted, then the attachment rate equals 0.3. We constructed a Beta regression with mixed effects that can be used to analyse percentage data. We used the ‘glmmTMB’ package to establish a random structure, with model selection performed with the ‘AICctab’ function from the ‘bbmle’ package. Parasite isolate was used as a random effect, and group, temperature and dryness as fixed effects. Analysis of variance is not recommended for Beta models, hence we used the ‘Analysis-of-variance-like’ function for factor terms ‘Joint_tests’ from the ‘emmeans’ package.

## 3. Results

### (a) Infection experiment

**Figure 3.**
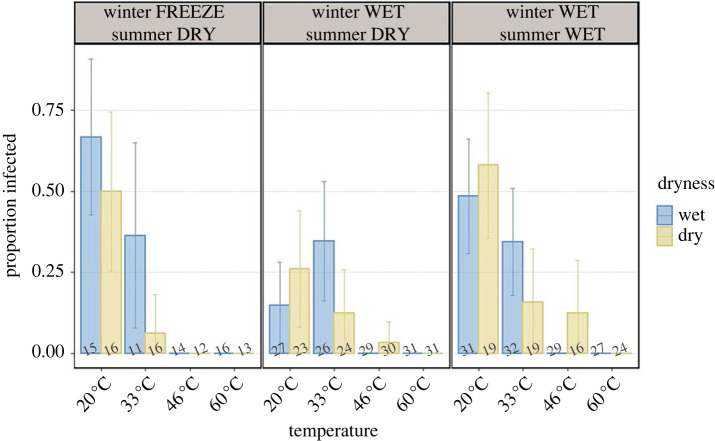
Parasite infectivity per group, temperature and dryness condition. Error bars represent 95% s.e. The numbers under the bars are the sample size. Winter FREEZE summer DRY—FD group; winter WET summer DRY—WD group; and winter WET summer WET—WW group. (Online version in colour.)

Out of 820 *Daphnia* individuals, 555 survived past the time that infection was visible, thus 265 individuals (including five controls) were excluded from further analysis. Of those 555 individuals, 88 became infected, 452 were exposed to the parasite but did not become infected, and 15 were healthy unexposed controls. No infected individuals were found in any of the 60°C treatment combinations. No infected individuals were found in the 46°C treatment under the wet condition (figure 3). Only three infections were found in the 46°C dry treatment, two in the WW group (BE-KN isolate) and one in the WD group (Tirtza isolate).

Infectivity was significantly affected by group (*p* = 0.01; table 2; figure 3) and temperature (*p* < 0.001). More precisely, infectivity decreased as temperature rose, from 41.2% at 20°C to 0% at 60°C. The only exception to this trend was the wet treatment in the WD group, where the infection rate rose with temperature from 14.8% at 20°C to 35.6% at 33°C. Group WD was the least infected, followed by groups WW and FD which had the highest infectivity. The interaction between group and temperature was nearly significant (*p* = 0.075), albeit at higher temperatures groups WD and WW had higher infectivity than group FD.

**Table 2.** Results of GLMs containing all tested variables. Bold typeface indicates significant effect (*p* < 0.05). LR stands for likelihood ratio. (Interactions were left in the model if *p*-value < 0.1.)

predicted variable	independent factor	d.f.	LR	*p*-value
infectivity	temperature	1	120.3	**<0.001**
	group	2	9.25	**0.010**
	dryness	1	1.38	0.240
	group × temperature	2	5.16	0.076
host survival (infected only)	temperature	1	0.25	0.621
	group	2	7.70	**0.015**
	dryness	1	0.06	0.811
	temperature × group	2	6.32	0.056
spore production	temperature	1	0.92	0.345
	group	2	1.52	0.445
	dryness	1	0.09	0.761
	temperature × group	2	7.72	**0.018**
clutch production (infected only)	temperature	1	0.06	0.776
	group	2	1.05	0.591
	dryness	1	2.58	0.085
	temperature × dryness	1	4.00	**0.030**

**Figure 4.**
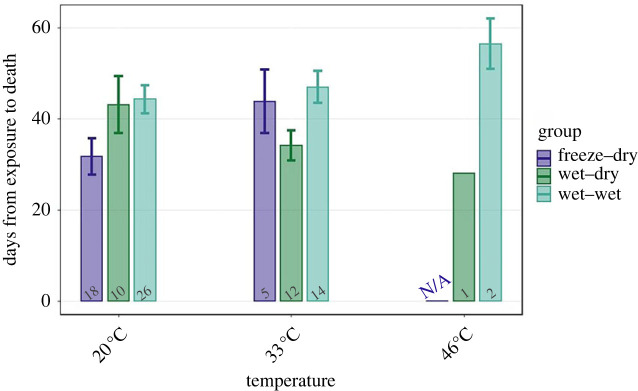
Longevity of infected hosts (days from exposure to death, i.e. virulence). The bars represent the means and error bars are s.e. The numbers under the bars are the sample size. (Online version in colour.)

Host longevity (virulence) was significantly affected by group (*p* = 0.015; table 2; figure 4), and the interaction between group and temperature was nearly significant (*p* = 0.056). Mean host longevity increased with temperature in group FD and slightly in group WW by 38% and 5.9%, respectively, including the wet and the dry treatments (31.7 to 43.8 days and 44.3 to 46.3 days, respectively). By contrast, mean host longevity decreased with temperature in group WD by 20.9% (43.1 to 34.1 days). Owing to the very scarce infections at 46°C, this treatment was not included in the abovementioned calculations.

**Figure 5.**
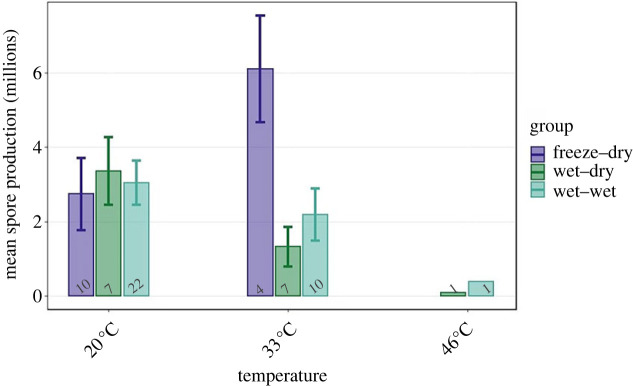
Parasite spore production upon host death. The bars represent the means and error bars are s.e. The numbers under the bars are the sample size. As the only significant explanatory variable is the interaction between temperature and group, we represented only them in the graph. (Online version in colour.)

Spore production was significantly affected by the interaction between temperature and group (*p* = 0.018; table 2; figure 5). More precisely, while in groups WD and WW spore production decreased with rising temperature, in group FD spore production rose with temperature. Yet, only groups WD and WW produced infections at 46°C, thus total spore production (young and mature spores) was 1.97 ± 0.28 million spores, while mean spore production for treatments subjected to 46°C was about fourfold lower, 0.52 ± 0.11 million spores.

**Figure 6.**
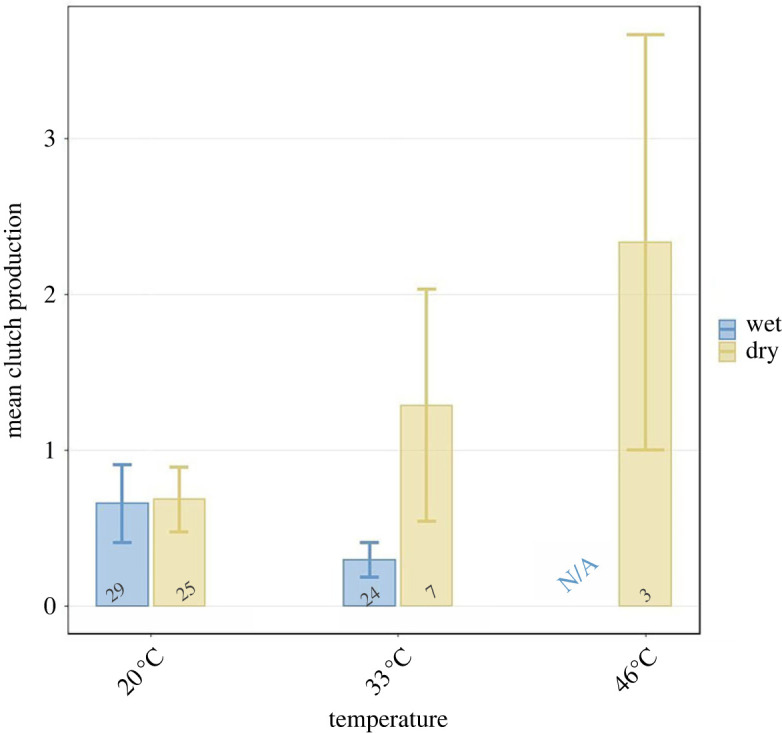
Mean number of host clutches produced. The bars represent the means and error bars are s.e. The numbers under the bars are the sample size. As the only significant explanatory variable is the interaction between temperature and dryness, we represented only them in the graph. (Online version in colour.)

Clutch production was only tested for infected *Daphnia*, and it was affected by the interaction between dryness and temperature (*p* = 0.030; table 2; figure 6). Specifically, when infected with bacteria from the dry treatment and higher temperatures, *Daphnia* produced on average more clutches than when infected with bacteria from wet conditions or lower temperatures (46°C and 33°C dry = 1.60 ± 0.64 offspring, 20°C dry = 0.68 ± 0.21 offspring, 33°C wet = 0.29 ± 0.11 offspring, 20°C wet = 0.65 ± 0.25 offspring).

### (b) Attachment experiments

**Figure 7.**
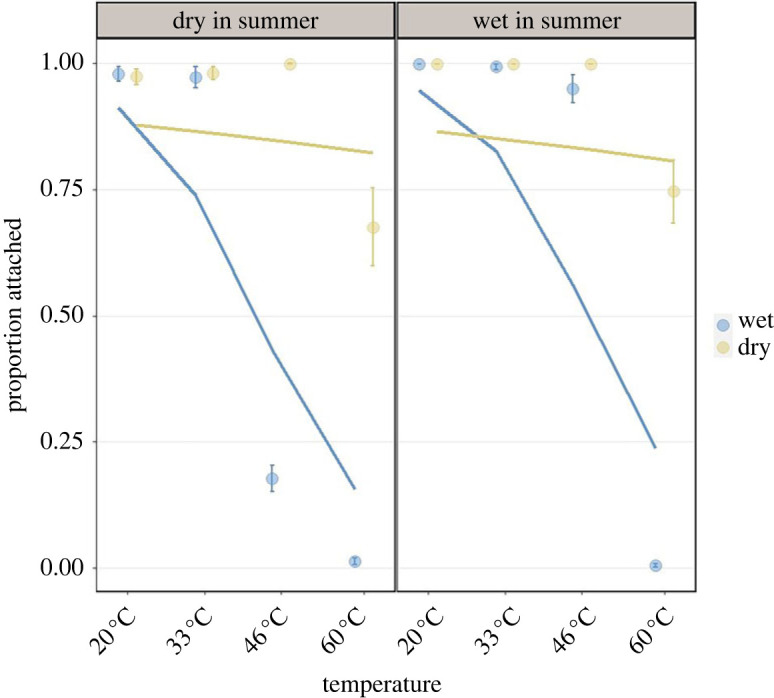
Mean proportion of spores that attached for each group, temperature and dryness condition. Circles represent the mean proportion of attachment and error bars are s.e. Curved lines are the predicting models. ‘Dry in summer’ contains groups FD and WD, and ‘wet in summer’ contains groups WW and FW. (Online version in colour.)

Attachment was successful in all treatments, except for spores from all groups in the 60°C wet treatment, where few or no attachments were detected (WW 60°C wet = no attachment, WD 60°C wet = 0–20 spores, FW 60°C wet = 0–10 spores, FD 60°C wet = 0–3 spores). The attachment rate decreased with temperature, and with the temperature × dryness interaction, the model predicts higher attachment in high temperatures of dried spores than wet ones (table 3; figure 7). We also found that the two ‘dry in summer’ groups (WD and FD) had similar attachment patterns, and the two ‘wet in summer’ groups (WW and FW) had similar attachment patterns. In other words, both ‘dry in summer’ groups had high attachment rate in the 46°C dry treatment and low attachment rate in the 46°C wet treatment (less than 0.25), whereas both ‘wet in summer’ groups had high attachment rate in both 46°C dry and wet treatments (see dryness × summer-group interaction in table 3). We compared the best model with all groups included to the best model with only the dry versus wet in summer division (hereafter referred to as summer-group). We found that summer-group is a better explanatory variable than the regular group division for the attachment data. Consequently, the best model chosen included temperature, dryness, summer-group, temperature × dryness interaction and dryness × summer-group interaction as explanatory variables (table 3; figure 7; models without a random structure had a better fit than models with a random structure).

**Table 3.** Lowest AICc model for attachment, showing ANOVA-like joint tests. Bold typeface indicates significant effect (*p* < 0.05). LR stands for likelihood ratio. (The ‘summer-group’ factor is either ‘dry in summer’ that pools groups WD and FD together, or ‘wet in summer’ that pools groups WW and FW together.)

predicted variable	independent factor	d.f.	*F*	*p*-value
attachment rate	temperature	1	534.8	<**0.0001**
	dryness	1	332.2	<**0.0001**
	summer-group	3	3.07	0.079
	temperature × dryness	1	373.8	<**0.0001**
	dryness × summer-group	3	8.82	**0.003**

## 4. Discussion

We investigated if environmental conditions during the off-host stage influence life-history traits of a bacterial parasite, and whether the parasite can adapt to the climatic conditions at its site of origin. We found that *P. ramosa* spores have reduced infectivity when resting at high temperatures. In addition, spores exposed to 60°C (dry and wet conditions) and 46°C (wet conditions) were unable to infect. Reproduction of infected hosts was affected by the interaction between temperature and dryness, insofar that hosts infected by spores exposed to 46°C produced on average more clutches than hosts exposed to other treatments. Spore production was significantly affected by the interaction between temperature and group, insofar that in the ‘wet but not frozen in winter’ groups (WD/WW) spore production decreased with rising temperature, whereas in the ‘frozen in winter’ group (FD) spore production rose with temperature. Host survival was also affected though not significantly by the interaction between temperature and group, yet significantly affected by group type alone. The attachment mechanism of *P. ramosa* spores was strongly affected by temperature, dryness and group type pooled by the summer conditions at origin (wet in summer-dry in summer). In agreement with the infection experiment, the attachment mechanism of dried spores was more resilient to temperature stress than that of wet spores. Spores subjected to 20 and 33°C had the highest rate of attachment and this rate decreased with temperature, insofar that when subjected to 60°C under dry conditions, spores could still attach, but at very low rates. Taken together, these results confirm our expectation that stress imposed on one life stage of the parasite can greatly affect subsequent stages, thereby emphasizing the importance of off-host stages in the study of host–parasite interactions.

We found that environmental conditions can differently affect parasitic traits that greatly impact host–parasite interactions and alter their dynamics. Temperature experienced during spore resting had a strong effect on every life-history trait of the parasite (including interactions with group or dryness), and consequently strongly influenced parasite fitness. Infectivity generally decreased with rising temperature, and the highest temperature reduced parasite fitness to zero. As a semelparous parasite, spore production upon host death (i.e. spore yield) represents the reproductive success of *P. ramosa*. Except for group FD, heated spores suffered from low reproductive success, which indicates that high temperatures can lead to less available spores and reduced opportunities for transmission. It has been suggested that intermediate to long infection periods can increase the yield [54–57]. However, hosts infected with spores subjected to 46°C lived longer in comparison to all infected *Daphnia* (figure 4). *Pasteuria ramosa* is a castrating parasite that uses the growth energy of its host as a resource, in order to redirect host resources from reproduction into body-mass investment [44,55,58]. This ability is subjected to an evolutionary arms race, where the host produces offspring earlier than normal (i.e. fecundity compensation) or regains fecundity after castration (i.e. castration relief), thereby harming parasite reproduction [44]. Thus, if parasite spores are incompetent or damaged, the host may produce more clutches. In this study, we found a significant effect of the dryness and temperature interaction on host clutch production. This may indicate that at higher temperatures, dried *P. ramosa* spores become damaged, and this damage is expressed in the form of increased host fecundity. Put differently, *D. magna* hosts are less likely to become infected by heat-stressed spores, and if they do, the parasite will not develop well in the host, the illness may be less harsh, and host fitness will be higher than by spores from less-stressed conditions.

To better understand why spores cannot infect their hosts under extreme conditions, we conducted the attachment experiments. Attachment to the host occurs prior to the penetration of the haemolymph and subsequent encounter with the host immune system [27,51]. This allowed us to determine if the spores are mechanically damaged and thus fail to attach to the host or the damage caused by extreme conditions is expressed later in the infection process. Our results show that the attachment mechanism of *P. ramosa* can endure extreme environmental factors. In all groups, dried spores were able to attach even after they had been exposed to 60°C, whereas wet spores were unable to attach, even though we still noticed activation of spores. Isolates from dry pools summer conditions (summer-dry group) had the lowest attachment rate at 46°C wet, as opposed to the isolates from wet conditions during summer (summer-wet group).

Based on the results of the infection and attachment experiments, we speculate that adaptation is needed to survive high temperatures under wet conditions [20], as observed in the closely related bacteria *Bacillus subtilis*, which is more dry-heat resistant than wet-heat resistant [20,59]. In *B. subtilis* resistance to wet-heat mainly depended on the spore's core water content, while resistance to dry-heat was related to DNA protection by small, acid-soluble spore proteins [59]. It is also consistent with prior studies of the effects of dry- and wet-heat on bacterial biofilm [60]. The decline in parasite performance (infectivity, virulence and attachment) with increasing temperature indicates that at high temperatures, *P. ramosa* spores are less sensitive to dry conditions than to wet ones. While this suggests that bacterial disease agents may survive a combination of droughts and heatwaves, whose risk owing to climate change is rising [19], it also suggests that wet environments subjected to heatwaves may have devastating effects on bacterial disease agents at the population and community levels.

In our experiments, the highest temperature used was 60°C. Although 60°C is an extreme temperature that is yet to be recorded in air in the southern area of the *P. ramosa* distribution range, surface temperatures can be higher than air temperatures, depending on vegetation coverage, clouds and water vapour [61]. The surface of dry pond sediments is often fully exposed to sunlight and thus may also reach very high temperatures. The parasitic isolates from the most heat-disturbed environment are Nitzanim and Tirtza (WD group). The Israeli coastline (Nitzanim) is characterized by Mediterranean climate, and thus summer is poor in precipitation, with high radiation, very hot weather and high dampness. In the desert (Tirtza), temperatures are even higher, but with high day–night fluctuations, no precipitation and low dampness. It is therefore quite surprising that no infections were recorded at 60°C using spores of isolates from the Israeli hot coastline and desert.

While it is less likely to experience three consecutive days of high temperatures in the wild, day–night temperature fluctuations are very common. Yet, we are unaware of studies that examined the effects of prolonged environmental conditions on *P. ramosa* spores and the long-term effects on the parasite, which is why we used uniform conditions to reduce variability. Following our fundamental work, future experiments should be conducted with different heat durations and fluctuations, as these were found to alter the outcome of host–parasite interactions [62,63]. This would allow differentiation between continuous heat conditions versus heatwave scenarios. It should be noted, however, that a study of *P. penetrans* that used day–night fluctuations and prolonged exposure showed results similar to those of our attachment experiment [64]. Therefore, it is likely that natural conditions and occasional heatwaves can cause similar effects under laboratory conditions. Heatwaves can persist for a few days, e.g. the heatwave that struck Israel in August 2021 and which caused some areas to experience more than 40°C lasted for at least six consecutive days [65]. During the same heatwave, near Tirtza reservoir (at Gilgal), the average air temperature was 44.7°C during the day and the maximal temperature was 45.6°C. Based on our results, at this temperature level relatively few spores are infective and thus less likely to cause disease. Recent models predict that owing to global warming, by the end of the century air temperatures during heatwaves in the Middle East are expected to reach 50°C [66]. Without adapting, *P. ramosa* from the Middle East would be at great risk. We do not know the effects of the same conditions on the host's resting eggs, but a reduction of host density surely poses a risk to the parasite, which in return can influence the composition of host populations and communities. In years of drought, freshwater ecosystems will have to withstand longer dry periods, and small ever-wet pools will evaporate [67]. Northern-Europe spores are adapted to survive at much higher temperatures than they are naturally exposed to, including desiccation, and thus will probably survive extreme weather events and drastic changes in the pool environment. Notwithstanding, our results show that even a temperature elevation (to 33°C) affected off-host stages from the European groups FD and WW.

We assumed adaptations to highly disturbed environments may cause a trade-off among parasite survival, infectivity and virulence. Hence, special adaptations would be found, particularly in extreme environments. For example, Rogalski & Duffy [68] examined the influence of solar radiation on six populations of *P. ramosa*, which originated from lakes with an ultraviolet radiation penetration gradient [68]. They found that populations from sunlight-exposed lakes were adapted to solar radiation, but there was a cost in the form of fewer spores produced after exposure. In our experiments, however, patterns of adaptations to extreme temperatures were not observed in highly disturbed groups. One possible explanation is that tolerance to extreme conditions may have not evolved owing to the existence of the spore bank, where spores can survive within the sediment for decades [26]. While the top layer of the sediment, which contains the newest *P. ramosa* spores, is exposed to all the weather conditions [69], the lower layers with older spores are less exposed and maintain lower temperatures [70], and are thus less sensitive to changes in the weather. This may allow the survival of the parasite after extreme weather events. Moreover, it is also possible that some host genetic lines are more vulnerable or tolerant than others to abiotic conditions. In this case, the prevalence of more heat-tolerant lines would depend on the weather conditions in previous generations. Unfortunately, we do not have details of the weather conditions when the original wild spores were harvested.

GxG interactions occur when different parasite genotypes cause the disease to express itself differently (e.g. infectivity, virulence) in the same host genotype, or vice-versa [71,72]. In this study, these interactions are expressed in the ‘group’ factor, which represents the habitats from where the *Pasteuria* isolates had originated. Not surprisingly, infectivity was significantly affected by group. Additionally, significant interactions between the group and temperature or dryness, suggest that GxG interactions occur only under certain circumstances, which are yet to be determined.

There is growing awareness worldwide in the urgency of dealing with climate change, including its impact on infectious diseases and the rise of epidemics. While gradual warming may allow many parasites to adapt owing to their short evolutionary timescales, extreme events appearing more frequently like heatwaves (i.e. pulse warming) [19] may cause drastic changes in all life stages of parasites, including increases or decreases in parasite survival and transmission [73]. To better understand how diseases are influenced by such extreme events, we must examine each aspect apart and all aspects together. This study of the direct effects of heat stress on the off-host stages of *P. ramosa* is the first step of such research of climatic influences on parasitic diseases. Our findings that heat stress may tamper with parasite fitness and transmission go beyond our particular *Daphnia*-*Pasteuria* system, as effects on parasite traits can cascade to other hosts and non-hosts, with far-reaching community-level effects on the outcome of host–parasite interactions [74]. In this respect, we should also consider the host response to gradual and pulse warming. In our system, *Daphnia* spp. are very corresponsive to warming, and the community of cladocerans can be rapidly modified owing to climate change and rapid warming [75,76]. For example, this can lead to a ‘dilution effect’, whereby susceptible hosts are less prevalent [73]. Therefore, a better understanding of the ecological and evolutionary dynamics and trajectories of host–parasite interactions is crucial for our efforts to unravel the basic mechanisms governing parasitic off-host stages.

## 5. Conclusion

In summary, subjecting off-host stages of endoparasites to extreme temperatures and dryness regimes can affect their survival and consequently the infection process. We argue that there is a twofold advantage to understanding the biology of off-host transmission-stage survival in the external environment [18] and suggest investigating it in other parasites as well. First, theoretical models show that the survival rate of transmission stages can strongly influence the epidemiology of disease and the evolution of its virulence [77,78]. This holds true for human parasites, our livestock and agricultural plants, and wildlife conservation. Second, ecological conditions for the survival of transmission stages (and also for disease vectors) are a key factor in determining the geographical distribution of parasites [79]. Ultimately, the finding that temperature and dryness affect the infectivity and virulence of the *Daphnia*-*Pasteuria* system may indicate that viability of environmental resting stages of various diseases and parasites could be affected by extreme weather events, which may in turn affect future disease outbreaks and disease dynamics. Moreover, with temperature and dryness interacting, and differently affecting host and pathogen traits, at different steps of the infection process, predicting how diseases will be impacted by increasing extreme weather events may need a deep understanding of the underlying mechanisms.

## Data Availability

Additional data are provided in Dryad: https://doi.org/10.5061/dryad.sqv9s4n7h [80]. Data is also provided in the electronic supplementary material [81].
